# Pediatric Acute Pancreatitis: A Seven-Year Cohort Study of Etiology, Severity, and Outcomes in Pakistan

**DOI:** 10.7759/cureus.106085

**Published:** 2026-03-29

**Authors:** Saima Mehmood, Dur E Shahwar, Wasif Ilyas Vohra, Paras Nisar, Sumera Sharafat, Memona, Sahar Malik Fayyaz, Kamran Sadiq

**Affiliations:** 1 Pediatrics and Child Health, Aga Khan University Hospital, Karachi, PAK; 2 College of Medicine, Jinnah Sindh Medical University, Karachi, PAK; 3 Pediatrics and Child Health/Pediatric Gastroenterology, Aga Khan University Hospital, Karachi, PAK

**Keywords:** acute pancreatitis, children, disease severity, etiology, outcomes

## Abstract

Objective: To identify the etiology, assess the severity, and evaluate the outcomes of acute pancreatitis (AP) in children admitted to a tertiary care hospital.

Methods: A retrospective medical record review was conducted at the Department of Pediatrics and Child Health of a tertiary care center from January 2018 to December 2024 after obtaining approval from the institutional ethical review board. The study included all children aged one month to 18 years with physician-diagnosed AP who presented during the study period. Medical records were reviewed to obtain relevant demographic and clinical information. The primary outcome measures included the etiology of pediatric AP, disease severity (mild, moderate, or severe), clinical presentation, incidence of complications during hospitalization, recurrence, and in-hospital mortality.

Results: In a study of 112 children with AP, 80 (71%) had mild disease, and 32 (29%) had moderate to severe cases. Idiopathic etiology was the most common, 79 (70.5%), whereas gallstone-related pancreatitis accounted for 9 (11%) in older children (11-18 years, n=81). No factors were independently linked to disease severity, and about 67 (60%) experienced recurrent pancreatitis (based on multiple documented episodes), though serious complications and mortality were rare.

Conclusion: In this pediatric cohort of AP from a low-resource country, idiopathic pancreatitis was the most common etiology. Most cases were mild, and systemic complications were rare, with no factors independently predicting disease severity.

## Introduction

Acute pancreatitis (AP) is an inflammatory condition of the pancreas that can also affect surrounding tissues and distant organ systems to varying degrees [1]. Pediatric pancreatitis has become an area of increasing focus in recent years, with several studies documenting a rise in the diagnosis of AP in children [2]. The incidence of pediatric AP has risen over the past two decades, now ranging from three to 13 cases per 100,000 population annually [3]. However, regional variations are evident. A study conducted in the UK reported an overall incidence of 0.78 per 100,000 children per year (95% CI: 0.62-0.96). Notably, Pakistani children in the same study exhibited a significantly higher incidence, at 4.55 per 100,000 children per year (95% CI: 2.60-7.39), which is approximately seven times greater than that observed in their White counterparts [4]. However, the study does not specify whether these children were first- or second-generation immigrants, limiting the interpretation of the relative contributions of genetic and environmental factors. The reported incidence of pediatric AP varies widely and likely reflects differences in study design, case ascertainment, and population characteristics.

Similarly, Cheng et al., in a Taiwan population-based study, documented a modest rise in the incidence of AP from 2.33 to 3.07 cases per 100,000 population between 2000 and 2013. This corresponds to an average annual increase of 0.05 cases per 100,000 population, with similar trends observed across both genders. The presentation of AP shows two peaks: the first occurs at ages 4 to 5, while the second begins in adolescence and continues into adulthood [5]. This age-related distribution may indicate varying underlying etiologies. Younger children are more likely to experience genetic or systemic causes, whereas older children tend to present with biliary or idiopathic pancreatitis. This discrepancy is thought to be influenced by a combination of genetic, environmental, and healthcare system-related factors. However, without data from resource-constrained environments, definitive conclusions cannot be drawn.

Park et al. identified key risk factors for AP in pediatric patients, including biliary tract disease, medication use, systemic disease, abdominal trauma, metabolic disorders, and inborn errors of metabolism. Etiologies of AP varied with age. In children under six years, causes were diverse, with idiopathic cases being rare. In children over 11 years, biliary causes were predominant, with gallstones accounting for most cases. Inborn errors of metabolism were exclusively seen in the 0 to two year age group [6]. Recent evidence suggests that the etiology of AP in children is multifactorial, with the most common causes including biliary disease, drug-induced pancreatitis, and idiopathic cases, with notable variability across different populations [7]. It is uncertain whether these patterns apply to Pakistan, where the challenges of trauma, prevalence of infectious diseases, and availability of advanced cardiac surgery differ significantly from those in high-income countries. To date, there is limited literature on the etiology, severity, and clinical outcomes of pediatric AP in South Asian countries, particularly in Pakistan.

This knowledge gap is particularly significant given Pakistan’s substantial burden of pediatric trauma and infectious diseases, limited healthcare resources, inconsistent access to specialized pediatric care, and the lack of routine genetic testing to identify hereditary causes of AP. These factors may influence both the causes and outcomes of the disease. Accordingly, this study aims to examine and describe the etiology, severity, and clinical course of AP in children at a tertiary care facility in Karachi, Pakistan.

## Materials and methods

This retrospective study was conducted at the Department of Pediatrics and Child Health, Aga Khan University Hospital, Karachi, Pakistan, after approval from the institutional ethics committee, and informed consent was waived due to the retrospective design. It included all children aged one month to 18 years admitted with AP between January 2018 and December 2024. Both first-episode and recurrent AP cases were eligible for inclusion. No formal a priori sample size calculation was performed due to the retrospective nature of the study, and all eligible patients during the study period were included consecutively to minimize selection bias.

Patients with chronic pancreatitis (CP) were excluded, defined as the presence of irreversible structural pancreatic changes (e.g., ductal abnormalities, calcifications, or fibrosis) with or without persistent exocrine or endocrine insufficiency. Patients with postoperative pancreatitis and cases with incomplete medical records insufficient to confirm diagnosis, etiology, or outcomes were also excluded.

For patients with recurrent AP (≥2 distinct episodes of AP separated by at least three months), only the index admission during the study period was included in the analysis. Cases were classified as first-episode or recurrent pancreatitis based on documented prior episodes in the medical record.

AP was defined as the presence of at least two of the following: (1) abdominal pain compatible with pancreatitis, (2) serum amylase or lipase ≥3 times the upper limit of normal, or (3) imaging findings suggestive of pancreatitis, including pancreatic edema, peripancreatic fat stranding, or necrosis on ultrasound (US), CT, or MRI [3].

Severity was classified according to the revised Atlanta classification. Although originally developed for adults, the revised Atlanta classification has been widely applied in pediatric studies for severity stratification: mild (no organ failure or complications), moderately severe (transient organ failure <48 h or local complications) [8], or severe (persistent organ failure >48 h), with organ failure defined using the Sequential Organ Failure Assessment (SOFA) criteria for cardiovascular, renal, or respiratory systems [9]. A pediatric-specific SOFA (pSOFA) score was not used.

Idiopathic pancreatitis was defined as AP in which no identifiable cause was established after a standard diagnostic evaluation including clinical history (alcohol use and medication review), laboratory testing (including serum triglycerides and calcium levels), and biliary imaging with transabdominal ultrasonography, with additional imaging such as magnetic resonance cholangiopancreatography (MRCP) or endoscopic ultrasound (EUS) performed when clinically indicated [10].

Demographic, clinical, laboratory, radiographic, and in-hospital outcome data (including complications, recurrence, mortality, and length of stay) were collected from the medical records of patients diagnosed with AP. Complications were classified as local (e.g., pseudocyst, necrosis) or systemic (e.g., organ failure). Laboratory results obtained within 48 hours of initial hospital admission were documented following approval of the ethics committee. Only de-identified data were used, and access was restricted to authorized research team members.

Descriptive statistics, such as means and standard deviations, were used to summarize continuous variables, including age, BMI, and hospital stay duration, for both the mild AP and moderate-to-severe AP groups. Frequencies and percentages were calculated for categorical variables such as gender, clinical presentations (e.g., abdominal pain, vomiting), and etiologies of AP. The chi-square test was applied to assess the distribution of etiologies and clinical presentations between the severity groups. An independent samples t-test was used to compare continuous variables between the groups.

The multivariable logistic regression model was limited to three clinical predictors, including age, serum lipase (per 100 U/L increase), and biliary etiology, due to the small sample size of moderate-to-severe AP cases. Variables were selected based on clinical relevance and univariable analysis. Missing data were handled using complete case analysis. Firth’s bias-reduced logistic regression was also employed to perform sensitivity analysis. Results are presented as odds ratios with 95% CI. All statistical tests were two-sided, with significance set at a p-value <0.05. Statistical analyses were performed using Stata version 17.0 (StataCorp LLC, College Station, TX, USA).

## Results

**Figure 1:**
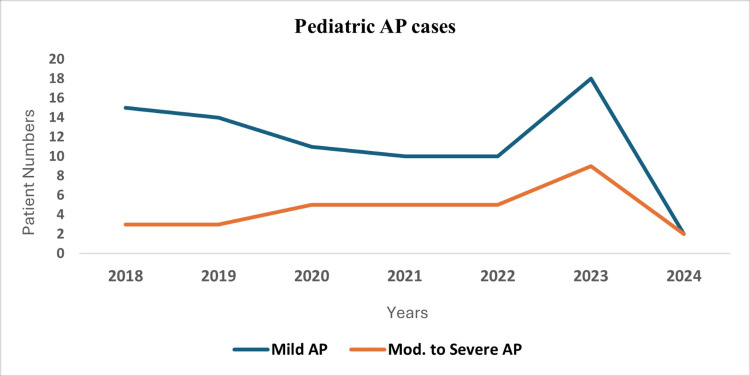
Annual distribution of mild and moderate-to-severe AP among children AP, acute pancreatitis

Among 112 children aged one to 18 years admitted with AP, 80 (71%) had mild disease and 32 (29%) had moderate-to-severe AP according to the Atlanta Classification System. The hospitalization trends throughout the study period are illustrated in Figure 1. Admissions for mild AP decreased over the years, while cases of moderate-to-severe AP showed fluctuations, reaching a peak in 2023.

Baseline characteristics by disease severity

Table 1 summarizes the demographic characteristics, clinical presentation, and laboratory findings of children diagnosed with AP stratified by disease severity. Children with mild AP had a mean age of 11.8±4.2 years and a mean BMI of 19.3±6.5, while those with moderate-to-severe AP had a slightly higher mean age (12.2±3.7 years) and a similar BMI (19.1±5.1). Males predominated in the mild AP group (47, 58.8%), whereas females were more frequently represented in the moderate-to-severe group (20, 62.5%).

**Table 1:** Demographic, clinical, and laboratory characteristics of children with AP stratified by disease severity AP, acute pancreatitis; TLC, total leukocyte count

Characteristics	AP status (N=112)	
	Mild AP (n=80)	Moderate to severe AP (n=32)
Age (years)	11.8±4.18	12.15±3.70
BMI (kg/m^2^)	19.27±6.52	19.12±5.11
Length of stay (days)	4.17±2.79	4.81±5.37
Amylase (I.U/L)	658.91±752.79	596.37±641.89
Lipase (U/L)	1113.49±1188.367	1021.43±1284.71
Calcium (mg/dL)	8.94±1.41	8.91±0.88
Potassium (mmol/L)	4.06±0.54	4.07±0.64
Bicarbonate (mmol/L)	22.73±3.84	22.70±3.52
TLC (x10^9^/L)	15.91±27.55	13.97±5.78
Creatinine (mg/dL)	0.58±0.33	0.60±0.23
Vitals heart rate (beats/min)	99.78±20.65	97.93±21.25
Vital respiratory rate (breaths/min)	24.06±9.45	23.5±6.01
Vitals temperature (C)	36.75±0.66	36.87±0.35
Vitals SPO_2 _(%)	98.95±1.07	98.75±1.43

**Figure 2:**
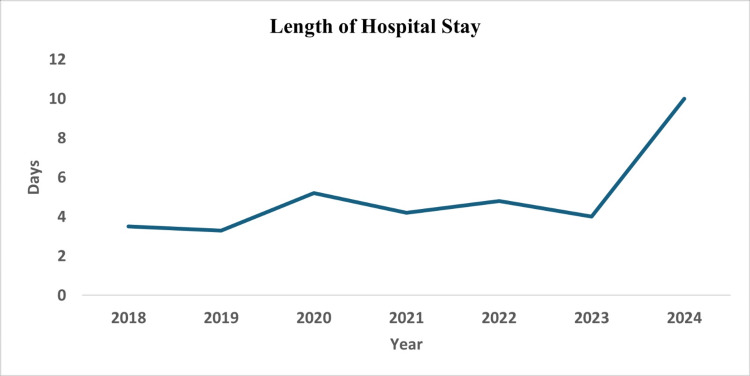
Trends in the length of hospital stay among children diagnosed with AP between 2018 and 2024 AP, acute pancreatitis

Abdominal pain with vomiting was the most common presenting symptom in both groups. Length of hospital stay was slightly longer for children with moderate-to-severe AP compared with those with mild AP (mean±SD 4.81±5.37 vs. 4.17±2.79 days, respectively), consistent with the greater clinical severity in the moderate-to-severe group (Figure 2).

Serum amylase and lipase levels were numerically higher in the mild AP group. Serum electrolyte values, creatinine, total leukocyte count, and presenting vital signs did not differ significantly by disease severity.

Etiology of acute pancreatitis

The distribution of potential causes of AP based on the severity of the disease is highlighted in Table 2. Idiopathic pancreatitis is the most common cause overall, accounting for 53 (66.3%) of mild AP and 26 (81.3%) cases of moderate-to-severe AP. Other causes, such as hyperlipidemia, autoimmune factors, and others, are less common.

**Table 2:** Distribution of AP etiologies among children according to severity status and age groups Values are presented as n (% within column). AP, acute pancreatitis

Etiology	Disease severity		Age group	
	Mild AP (n=80)	Moderate/severe AP (n=32)	0-10 years (n=31)	11-18 years (n=81)
Autoimmune	3 (3.75%)	0 (0%)	1 (3.2%)	2 (2.4%)
Biliary disease	7 (8.75%)	2 (6.25%)	3 (9.6%)	6 (7.4%)
Gallstones	7 (3.75%)	3 (9.3%)	1 (3.2%)	9 (11.1%)
Hyperlipidemia	5 (6.25%)	0 (0%)	3 (9.6%)	2 (2.4%)
Idiopathic	53 (66.25%)	26 (81.25%)	21 (67.7%)	58 (71.6%)
Others	5 (6.25%)	1 (3.13%)	2 (6.4%)	4 (4.9%)
Total	80 (100%)	32 (100%)	31 (100%)	81 (100%)

When stratified by age, etiologic distribution differed significantly between children aged 0-10 years and 11-18 years (χ²=12.09, df=5, p=0.034). Gallstone-associated pancreatitis was more common in older children, while idiopathic pancreatitis remained the predominant etiology across both age groups (Table 2).

In multivariable logistic regression adjusting for age, body mass index (BMI), serum amylase, and serum lipase levels, no etiology or covariate was independently associated with moderate-to-severe AP (Table 3).

**Table 3:** Multivariable logistic regression analysis of etiological factors associated with AP AP, acute pancreatitis; BMI, body mass index

Variable	Coefficient	Standard error	z-value	P-value	95% CI
Biliary disease	-0.4850227	1.612324	-0.30	0.764	(-3.64512, 2.675075)
Gallstones	0.667174	1.466087	0.46	0.649	(-2.20603, 3.540651)
Idiopathic	0.8043606	1.177582	0.68	0.495	(-1.503658, 3.112379)
Age	-0.0054525	0.0802379	-0.07	0.946	(-0.1627158, 0.1518108)
BMI	-0.0033256	0.0494418	-0.07	0.946	(-0.1002297, 0.0935784)
Amylase	-0.000306	0.0005802	-0.53	0.598	(-0.0014431, 0.0008311)
Lipase	-0.0000818	0.0003187	-0.26	0.797	(-0.000542, 0.0007065)
Constant	-1.261422	1.481981	-0.85	0.395	(-4.165875, 1.643031)

Outcomes, recurrence, systemic complications, and diagnostic findings

All patients included in the study were discharged alive, with no in-hospital mortality; therefore, comparative analysis of discharge outcomes was not performed. The frequency of recurrent pancreatitis did not differ by gender (female 61.0% vs. male 60.4%; χ²=0.005, p=0.945) or by disease severity (χ²=0.03, p=0.854).

Systemic complications were uncommon and did not show a significant difference between mild and moderate-to-severe AP (χ²=4.23, df=4, p=0.376). Most children had no documented systemic complications (72.5% in mild AP vs. 71.9% in moderate-to-severe disease). Hypertension was the most frequent complication, 10 (12.5%) in mild AP and 5 (15.6%) in severe AP, while sepsis and renal failure were rare.

Imaging findings on chest radiograph, US, CT scan, and endoscopic retrograde cholangiopancreatography showed no significant association with disease severity (all p>0.25).

## Discussion

This study aimed to describe the clinical profile, etiologic distribution, severity patterns, and outcomes of pediatric AP. The principal findings were a predominance of idiopathic cases and a recurrence rate of 60%, which is substantially higher than reported in most pediatric cohorts, where recurrence typically ranges from approximately 15% to 35% [11,12]. One reason could be differing definitions of acute recurrent pancreatitis (ARP/RAP) across studies. Many require at least two separate episodes with full clinical resolution, but variations in time intervals, imaging confirmation, and enzyme normalization criteria can affect reported recurrence rates [3]. Beyond definitional variation, context-specific healthcare factors likely play a central role. In our setting, the combined effects of definitional differences, referral bias, delays in seeking help, a lack of advanced diagnostic tools (such as genetic testing or advanced imaging), and variable access to specialized pediatric care may predispose patients to a higher recurrence rate.

Furthermore, fragmented follow-up systems and challenges with follow-up can lead to incomplete resolution and inadequate secondary prevention, significantly increasing the risk of recurrence. On the other hand, high-resource regions often report lower rates of idiopathic cases and milder disease, likely because of earlier detection, comprehensive diagnostic workup, and more uniform management protocols. The increased recurrence observed in our group may also stem from delayed diagnosis, inadequate management of underlying issues, or limited capacity for ongoing follow-up care.

Abdominal pain accompanied by vomiting was the predominant presenting symptom, mirroring findings from recent pediatric series where abdominal pain is present in the vast majority of cases (>90%) [13]. Other symptoms occurred less frequently, as described in prior literature [14]. Our regression analysis did not identify any factors, including etiology, as independently associated with moderate-to-severe AP. However, prior pediatric studies have reported biliary issues as a major cause, noting that recurrent AP and pseudocyst formation are more frequent in ARP [15]. These similarities suggest that children with biliary pancreatitis may constitute a unique subgroup that is at greater risk for experiencing more severe illness or complications [16].

A comparison with recent studies shows both agreement and differences. Volkan et al. reported that nearly one-third of children experiencing their first episode of AP progressed to ARP, and a considerable minority developed CP during follow-up [14]. A recent regional report highlighted a significant occurrence of biliary and structural-related issues, such as choledochal cysts, which were linked to more severe disease and a higher rate of recurrence [17]. In our cohort, we found no correlation between serum enzyme levels (amylase/lipase) and disease severity, which aligns with recent literature indicating that enzyme magnitude does not reliably predict severity in pediatric pancreatitis [18,19]. Additionally, imaging modalities such as US, CT, CXR, and ERCP showed no significant association with AP severity. This is consistent with recent evidence showing that although imaging is essential for diagnosis and detecting complications, early imaging has limited value in predicting disease severity in pediatric AP [20].

No deaths were reported within our cohort, and regional evidence reflects similarly low mortality. Kumar et al. reported a mortality rate of 1.6% in their study conducted in India [21]. In contrast, local data suggest that there is no recorded mortality among children with AP. Although the overall prognosis for this condition is generally favorable, it is important to note that the risk of mortality, while low, remains clinically significant, particularly in cases classified as severe [22]. Hypertension was the most frequent clinical finding in our cohort. This may be explained by several factors, i.e., systemic inflammatory response and significant pain, both of which can activate the sympathetic nervous system and lead to transient elevations in blood pressure.

Several methodological challenges may impact our interpretation of the findings. The high proportion of idiopathic pancreatitis cases likely reflects limited access to advanced diagnostic methods, such as detailed imaging and genetic or metabolic testing. This limitation has also been acknowledged in recent studies conducted in resource-limited settings [20]. In our study, cases were classified as idiopathic when no etiology was identified based on the available diagnostic evaluation, and therefore, some cases may represent undetected causes rather than true idiopathic disease. As a tertiary care referral center, our sample is likely to include a higher proportion of recurrent or complicated cases. Consequently, this may lead to an inflated incidence of recurrence rates in comparison to what is typically reported in other pediatric cohorts [23].

This study's strengths include its systematic evaluation of pediatric AP in a tertiary care referral setting, allowing for detailed insight into clinical patterns and short-term outcomes. The use of severity stratification and standardized data collection enhances consistency and supports deeper analysis beyond descriptive reporting. Additionally, it provides valuable context-specific evidence from a resource-limited healthcare environment, filling a significant gap in pediatric pancreatitis literature and underscoring the findings' relevance for similar settings.

Our analysis did not include long-term monitoring; however, earlier research has shown that ongoing complications, particularly pancreatic insufficiency, can arise [24]. The long-term effects of CP, exocrine insufficiency, and endocrine dysfunction are still unclear. Further investigation into genetic predisposition, anatomical variations, and healthcare access barriers is needed to address the high rates of idiopathic and recurrent cases. In addition, the retrospective design of this study introduces the possibility of missing data and selection bias, and as a single-center study conducted in a tertiary-care setting, the findings may have limited generalizability to other settings. Given the small number of moderate-to-severe AP events, our multivariable logistic regression model may still be at risk of overfitting, which could contribute to null findings despite limiting the number of predictors and performing a Firth’s bias-reduced sensitivity analysis.

Despite these limitations, our study provides valuable insights into the clinical profile, etiology, and outcomes of pediatric AP in Pakistan, highlighting key areas for prospective research with standardized diagnostics, follow-up protocols, and incorporation of genetic testing and imaging.

## Conclusions

In our pediatric cohort, AP was primarily mild, with idiopathic cases being the most common cause across various age groups. There were no demographic, biochemical, etiological, or imaging factors that independently correlated with the severity of the disease. However, this may reflect limited statistical power rather than a true absence of correlation. Short-term outcomes were generally favorable, with low rates of systemic complications and no in-hospital mortality. These results highlight the clinical diversity of pediatric AP and the need for more accurate diagnostic testing to determine etiology. They also address ongoing challenges in predicting disease severity and recurrence in everyday clinical practice.
